# Eco-Friendly Estimation of Heavy Metal Contents in Grapevine Foliage Using In-Field Hyperspectral Data and Multivariate Analysis

**DOI:** 10.3390/rs11232731

**Published:** 2019-11-20

**Authors:** Mohsen Mirzaei, Jochem Verrelst, Safar Marofi, Mozhgan Abbasi, Hossein Azadi

**Affiliations:** 1 Environmental Pollutions, Grape Environmental Science Department, Research Institute for Grapes and Raisin (RIGR), Malayer University, Malayer 65719-95863 Iran; 2 Image Processing Laboratory (IPL), Parc Científic, Universitat de València, 46980 Paterna, València, Spain; 3 Grape Environmental Science Department, Research Institute for Grapes and Raisin (RIGR), Malayer University & Water Science Engineering Department, Bu-Ali Sina University, Hamedan 65178, Iran; 4 Faculty of Natural Resource and Earth Science, Shahrekord University, Shahrekord 8815648456, Iran; 5 Department of Geography, Ghent University, 9000 Gent, Belgium

**Keywords:** field spectroscopy, hyperspectral, heavy metals, grapevine, PLS, SVM, MLR

## Abstract

Heavy metal monitoring in food-producing ecosystems can play an important role in human health safety. Since they are able to interfere with plants’ physiochemical characteristics, which influence the optical properties of leaves, they can be measured by in-field spectroscopy. In this study, the predictive power of spectroscopic data is examined. Five treatments of heavy metal stress (Cu, Zn, Pb, Cr, and Cd) were applied to grapevine seedlings and hyperspectral data (350–2500 nm), and heavy metal contents were collected based on in-field and laboratory experiments. The partial least squares (PLS) method was used as a feature selection technique, and multiple linear regressions (MLR) and support vector machine (SVM) regression methods were applied for modelling purposes. Based on the PLS results, the wavelengths in the vicinity of 2431, 809, 489, and 616 nm; 2032, 883, 665, 564, 688, and 437 nm; 1865, 728, 692, 683, and 356 nm; 863, 2044, 415, 652, 713, and 1036 nm; and 1373, 631, 744, and 438 nm were found most sensitive for the estimation of Cu, Zn, Pb, Cr, and Cd contents in the grapevine leaves, respectively. Therefore, visible and red-edge regions were found most suitable for estimating heavy metal contents in the present study. Heavy metals played a significant role in reforming the spectral pattern of stressed grapevine compared to healthy samples, meaning that in the best structures of the SVM regression models, the concentrations of Cu, Zn, Pb, Cr, and Cd were estimated with R^2^ rates of 0.56, 0.85, 0.71, 0.80, and 0.86 in the testing set, respectively. The results confirm the efficiency of in-field spectroscopy in estimating heavy metals content in grapevine foliage.

## 1 Introduction

In-field spectroscopy provides a time and cost-efficient and accurate way to monitor plant stress [1–3]. These hyperspectral data are sensitive to small differences in plant features; i.e., plant disease [4–6], water content [7,8], biomass assessment [8,9], crops quantity and quality [10,11], species and varieties discrimination [12–14], and heavy metal stress [1,2,15].

Heavy metal contamination in food-producing ecosystems is considered to be a major environmental problem due to its potential hazard to humans and other organisms and due to the intention to protect the safety of food chains [16,17]. Within the selection of human food, grapes and their secondary products (wine, jam, juice, jelly, vinegar, grape seed oil, and raisins) play an important role. Therefore, the safety of vineyards in terms of heavy metals is a key factor in grape production and wine industries [17,18]. In viticulture areas, the excessive and prolonged usage of fertilizers and pesticides releases heavy metals (i.e., Cu, Zn, Cd, Pb, Cr, Ni, Hg, and As), which has been considered in many studies [16-20]. According to Milićević et al. [18] and Sun et al. [17], significant correlations occur between heavy metal concentration in soil, grapevine parts (leaf, skin, pulp, and seed), and wine. Alagić et al. [21] also concluded that the grapevine has some highly effective strategies involved in tolerance to heavy metal stress, which makes it an excellent plant species for phytostabilization purposes. Therefore, grapevine foliage monitoring can potentially demonstrate heavy metal concentration states in other parts of the plant and is also acknowledged to be a bio-indicator of heavy metals in the enclosing environment.

Heavy metal stress can produce some changes in plant morphological and biochemical characteristics [15]. This is because the leaf spectral response is mainly affected by plant structural and morphological characteristics; i.e., the leaf’s intracellular and extracellular structure, and biochemical parameters such as nitrogen, pigments, and water contents [22–27].

Usually, heavy metal concentrations are detected in plant samples by acid digestion–solvent extraction followed by hydride generation atomic absorption spectrometry [28,29]. This tedious approach is expensive and destructive. Alternatively, by modeling the relationships between the heavy metal concentrations and foliar spectral characteristics, these concentrations can be efficiently estimated without using any chemical solvents. Therefore, by analyzing leaf spectral data, it becomes possible to investigate the biochemical and morphological changes caused by heavy metal stress [15,30]. It should be noted that in-field spectroscopy is one of the most attractive fields in remote sensing studies and can record specific spectral data to any object such as fingerprints [31,32]. Hyperspectral sensors can be used in the in-field spectroscopy process and so provide a framework for spectral reflectance acquisition in hundreds of narrow and contiguous bands/wavelengths [24,26]. Accordingly, it is expected that a plant being exposed to heavy metal stress will lead to subtle differences in the spectral curve as opposed to a healthy plant. These differences mainly occur in the visible and near-infrared regions of the electromagnetic spectrum [33].

Several studies have made specific use of the application of crop spectral characteristics through in-field spectroscopy data and multivariate statistical analysis to promote the prediction of heavy metal content in plant samples. For instance, Font et al. [28] and Font et al. [29] applied visible and near-infrared spectroscopy and the modified partial least squares (PLS) method to forecast metal content in prostrate amaranth and rice, with determination coefficients of 0.63 and 0.65, respectively. In another study, Rosso et al. [34] examined the spectral and physiological responses of *Salicornia virginica* to heavy metal (Cd and V) stress in laboratory conditions. The potential of in-field spectroscopy to detect heavy metal contents was also investigated by Ni et al. [35], Gu et al. [36], Liu et al. [37], Liu et al. [38], and Li et al. [39] in the case of dominant plants in the Poyang lake wetlands, *Brassica rapa chinesis*, rice, *Phragmites australis*, and vegetables, respectively.

It is worth noting that in-field spectroscopy delivers a large amount of spectral data, whereby each of the wavelengths may be associated with one of the plant parameters [40]. Therefore, identifying optimal wavelengths to monitor any parameter—e.g., heavy metal concentrations—is an important step in applying these data [41]. In this regard, the usage of multivariate statistical techniques such as the PLS method [14,40,42,43], multiple linear regression (MLR) [41,44,45], and support vector machines (SVM) [12,40,46] can help with feature selection, data reduction, and modelling the existing relationships between hyperspectral data and plant characteristics. Many studies have also taken advantage of spectral indices to minimize atmospheric and background disturbances and illustrate plant characteristics [3,15,30,45,47]. These indices are mathematical spectral transformations of two or more bands designed to enhance the spectral response of vegetation properties [12,40,46]. Hence, spectral indices calculated from foliar reflectance data may reveal the biochemical and physiological properties of leaves, which may be responsible for monitoring plant characteristics [46]. Despite the proven performance of in-field spectroscopy in estimating heavy metal contents in plants, to the best of our knowledge, such a study has never been employed on grapevines leaves.

Altogether, this study was designed with the following goals: i) developing hyperspectral libraries of healthy and heavy metal-stressed grapevine leaves (Vitis vinifera cv. Askari, as a common grapevine variety in Iran) by using full range in-situ spectroscopy (350-2500 nm), ii) evaluating the potential of in-field spectroscopy for estimating heavy metals (Cu, Zn, Pb, Cr, and Cd) concentrations in grapevine foliage, iii) investigating two types of hyperspectral data (wavelengths vs. spectral indices) and identifying the most appropriate features to estimate each studied metal in grapevine foliage, and iv) comparing the performance of SVM and MLR algorithms in modeling the relationships between the foliar spectral response and heavy metal concentrations.

## 2 Materials and Methods

### 2.1 Pollutant Exposure Experiments

Since experience in the evaluation of in-field spectroscopy when estimating heavy metal contents in grapevine leaves is lacking, we chose to conduct this research in a laboratory-controlled environment. Therefore, treatments for heavy metal stress were applied to grapevine seedlings. For this purpose, five treatments at varying levels of Cu, Zn, Pb, Cr, and Cd were considered, and in each treatment, four repetitions were carried out (a total of 84 grapevine seedlings were examined).

**Figure 1:**
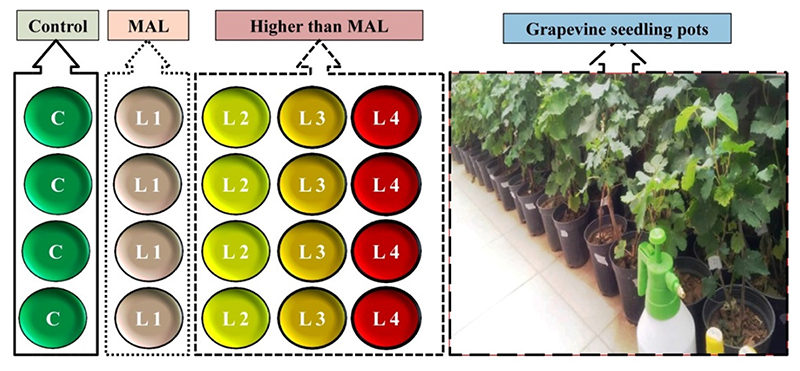
Schematic design of the treatment for each studied metal (C: control, L1 to L4: level 1 to level 4 stressed, MAL: maximum allowed level) and image of applied grapevine seedling pots.

It should be noted that the objective of this experiment was not to determine the sensitivity of grapevine to pollutants. We only intended to add heavy metal contents to the grapevine to compare its spectral differences with healthy leaf samples. The common grapevine variety in the study area is *Vitis vinifera cv*. *Askari*; all seedlings belonged to this variety to eliminate the effect of variety change on spectral characteristics [43,48]. Experiments were conducted outdoors in full sun between March and September 2018. Each grapevine seedling sample was placed in an individual plastic pot (length and width 25 cm × 10 cm) and was randomly divided amongst the studied treatments. The seedlings were two years old, and their height at the beginning of the experiment was between 20 and 30 cm. All seedlings were in the same conditions in terms of soil, pot size, sunlight exposure, watering, temperature, and humidity. In Figure 1, a schematic of the applied treatments is displayed. The first treatment served as a control to monitor the potential effects of soil, water, and air on the transfer of heavy metals to grapevine seedlings. In the second treatment, the maximum allowed level (MAL) of Cu, Zn, Pb, Cr, and Cd in irrigation water provided stress to the seedlings. All contamination levels were increased in the third, fourth, and fifth treatments as two, three, and four times the metal MALs in irrigation water, respectively. A stress program was applied to treatments 2–5 by dissolving the metal salts (nitrate form) in irrigation water. Salt metals have a high solubility, resulting in the absorption of the metals by plant organs [34]. According to the Iranian Water Quality Standard (IWQS), the MALs for Cu, Zn, Pb, Cr, and Cd in irrigation water are 200, 2000, 100, and 10 mcg/l, respectively. Seedlings were examined for a period of seven months, and they were stressed during each month (a total of seven stresses were applied). At the end of the stress period and before the beginning of the fall season (September 2018), a spectrophotometric analysis of grapevine seedlings leaves was applied.

### 2.2 Spectra Acquisition

At least five leaves of each seedling pot were collected for spectroscopy measurements (a total of 420 spectra samples were taken), and afterwards, individual reflectance spectra were measured by pot. In this study, the grapevine foliar spectral reflectance was measured using the ASD FieldSpec 3 spectroradiometer in the full range (350–2500 nm). This instrument is supported by three separate spectrometers (first: 350–975 nm, second: 976–1770, and third: 1771–2500 nm). The ASD spectral resolutions in the range of 350–1000 nm and 1000–2500 nm are 3 and 10 nm with sampling intervals of 1.4 and 2 nm, respectively. In accordance with Kumar et al. [49], the electromagnetic spectrum in the range of 350 to 2500 nm can be classified into four regions including visible (VIS), red-edge region (RDE), near-infrared (NIR), and mid-infrared (MIR), with ranges of 350~700, 680~750, 700~1300, and 1300–2500 nm, respectively. We performed the spectroscopy experiment in a fully dark room in order to reduce the effect of wind, water vapor, temperature, and other environmental disturbance [12]. In this study, each spectral sample was recorded in 100 automatic replicates. Then, we applied the ViewSpect version 6.0 in order to convert spectral curves into test files and analyze them by statistical software.

For each sample, the reflectance spectrum was recorded at 2151 wavelengths (350–2500 nm), which gave a large amount of data, not all of which may be useful for the study purpose. Therefore, in this study, 32 spectral indices were calculated to evaluate their ability to estimate heavy metal contents. The spectral indices which are used in this study were calculated based on the method indicated by Mirzaei et al. [12], although no specific spectral indices exist to detect heavy metal contamination [1]. Table 1 shows the indices that have demonstrated sensitivity in previous studies to small differences in plant characteristics [12,46,50].

### 2.3 Heavy Metal Laboratory Analysis

The leaves of each pot were placed in polyethylene bags and converted separately in the laboratory after obtaining the foliar reflectance spectra. The leaf samples were dried for 24 hours in an oven at 45 °C to achieve a constant weight [16]. The samples were powdered and stored for further analysis with a stainless-steel mill. We then digested one gram of each grapevine sample with HNO3 + HClO4 (3:1 v/v) for about 4 hours at a low temperature (about 40 °C) [51]. All digested samples were then diluted and filtered to 25 ml. Finally, a Graphite-Furnace Atomic Absorption Spectrophotometer (GA-AAS, Model: Analytik Jena, Germany) was used to analyze all samples in triplicate. The concentrations of heavy metal samples were expressed as dry weight (DW) mg/kg. The device detection limits for Zn, Cu, Pb, Cr, and Cd were 0.008, 0.025, 0.01, 0.04, and 0.009 mg/kg, respectively. Based on the analysis, the relative standard deviation accuracy was less than 9%. To evaluate the accuracy of analytical techniques, a spike-and-recovery analysis was performed. Post-analyzed samples were accentuated and homogenized with varying amounts of standard metal solutions. The recovery ranged from 90% to 108% of the spiked sample [52].

### 2.4 Feature Selection/Partial Least Squares (PLS)

In summary, the dependent variables were the contents of Cd, Cr, Cu, Pb, and Zn in grapevine leaves, while the independent variables were wavelengths (count: 2151) and spectral indices (count: 32). However, a large number of independent variables can reduce the performance of the relationship modelling between spectral data and metal contents. To mitigate this, we needed a feature selection process to identify optimal features (wavelengths and spectral indices) to forecast the concentration of each metal, individually. Also, before applying statistical operations, it is recommended to scale each variable linearly to the same standard range, especially in the machine learning methods [40,53]. The values of wavelengths, spectral indices, and heavy metal concentrations were therefore scaled to the range between 0 and 1, as follows:

(1) $$N_{i}=\frac{x_{i}-x_{\min}}{x_{\max}-x_{\min}}$$

where *N_i_* is the normalized value, *x_i_* is the original data, and *x*_min_ and *x*_max_ are the minimum and maximum of each variable’s percentages, respectively.

Given the high-dimensional spectral dataset, the use of multivariate statistical analysis is an appropriate solution for achieving optimal features to estimate each metal. PLS is a robust and well-known statistical analysis in relation to hyperspectral data that has shown acceptable performance in many studies [12,40]. This statistical analysis method generates some new components instead of using existing inputs, based on the least square regression. Unlike principal components analysis (PCA), PLS considers response variables in the data reduction process [54]. Fitting a regression model between input and output variables, high collinear spectral data, and the high processing speed are the other advantages of the PLS method. The PLS-developed components are capable of explaining community variance by a simpler structural mechanism. Accordingly, the importance of each input variable is realized by its factor load in each component [12]. We therefore selected optimal independent variables (wavelengths or spectral indices) based on the maximum factor load in each developed PLS component. These variables were considered to be the most representative of the related components. Based on the PLS results, the optimal wavelengths and indices were identified and introduced to the next step (modelling). Wold et al. [55] has provided more information about the assumptions and applications of the PLS.

### 2.5 Modelling the Relationship Between Spectral Data and Heavy Metal Contents

After the identification of the optimal wavelengths and relevant indices by the PLS, two types of modelling algorithms (SVM and MLR) were applied to estimate heavy metal concentrations based on hyperspectral data. To assess the estimation performance of each model, two goodness-of-fit indicators—specifically, the coefficient of determination (R^2^) and root mean squared error (RMSE)—were used [40]. All achieved data in this study were randomly separated into two sections: 70% as training data and 30% as testing data. As such, the performance of each developed model was individually reported for training and testing sets.

#### 2.5.1 Support Vector Machine (SVM)

SVM is a nonparametric learning algorithm for regression and classification goals and for hyperspectral data mining [56–58]. In the SVM procedure, the n-dimensional input vectors are conveyed into a high-dimensional feature space, and consequently, the optimal separating hyperplanes are developed [59]. Here, the SVM regression algorithm was used in multiple scenarios and designs to gain the best performance for modelling the relationship between the in-field hyperspectral data and the measured heavy metal concentration in grapevine leaves. To this end, the input vectors were linked to the outputs with a kernel function [12]. Regression SVM-type 1 with different kernel functions—i.e. radial basis functions (RBF), polynomials, and a sigmoid shape—was applied. In order to achieve an optimal training constant, V-fold cross validation was used, and kernel function parameters (coefficient, gamma, and degree) were altered to give a high-performance score [60]. More details about the assumptions and structure of SVM are provided by Stitson et al. [59] and Cristianini and Shawe-Taylor [61].

#### 2.5.2 Multiple Linear Regressions (MLR)

MLR is a parametric regression algorithm that attempts a relationship model between two or more independent variables and a response variable with a linear fitting. It has the capacity to select appropriate input data. In this study, the forward selection method of MLR was applied to increases the R^2^ value by adding an independent variable [40]. The Durbin–Watson statistic was applied to test autocorrelation in the residuals from statistical regression analysis. Durbin–Watson values close to 2 (1.5-2.5) indicate that there is no autocorrelation detected in the samples. Additionally, in order to detect multicollinearity in regression analysis, thw variance inflation factor (VIF) was considered (VIFs exceeding 10 are signs of serious multicollinearity) [62,63]. The general form of the MLR equation is as follows:

(2) $$\text{HMC}\;=\;{\text{a}}_{0}+a_{1}LM_{1}+a_{2}LM_{2}+\;...+a_{n}LM_{n}$$

where HMC is the heavy metal concentration in grapevine leaves, a _(i = 0,1,...,n)_ are the parameters generally estimated by least squares, and X _(i = 1,2,...,n)_ are the independent variables (i.e., wavelengths and spectral indices).

## 3 Results and Discussion

### 3.1 Reflectance Spectra of Healthy and Stressed Leaves

**Figure 2:**
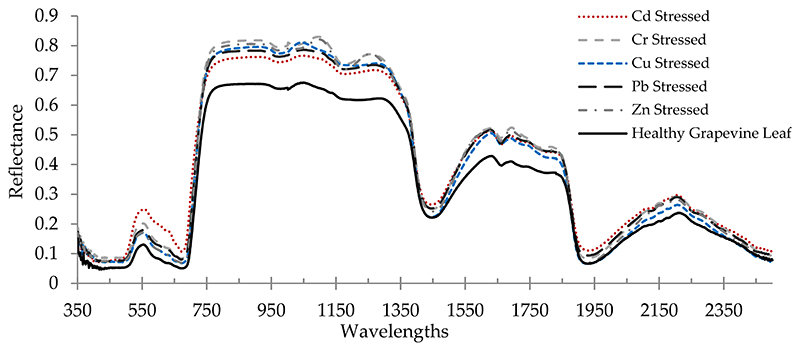
Average reflectance spectrum of healthy grapevine leaves vs. the heavy metal-stressed grapevine leaves (from 350 to 2500 nm).

The average reflectance spectrum of a healthy grapevine vs. stressed grapevine leaves due to heavy metal stress is shown in Figure 2. In the VIS region, the light absorption rate of the stressed grapevine was drastically decreased. This is due to the fact that the spectral characteristics of plants in this region are regularly motivated by pigments [64,65]. Accordingly, this suggests that heavy metal stress reduced pigment contents. Various spectral characteristics between healthy and stressed leaves can also be observed in the RDE, NIR, and MIR regions (Figure 2). As Vogelmann [66], Slaton et al. [23], and Strever [67] stated, plant pigments do not absorb the light in the NIR and MIR regions; therefore, the plant leaf reflectance is significantly increasing in these regions. Additionally, the spectral characteristics of plant leaf in the NIR and MIR regions were changed by structure/morphology and water contents, respectively [54]. According to Figure 2, in the NIR and MIR regions, a lower reflectance was observed in healthy grapevine leaves as opposed to the stressed grapevine. Although other driving variables such as structural parameters and water contents were not measured in this study, it can be concluded that the stress caused by heavy metals had a significant effect on the leaf optical properties.

### 3.2 Correlation Coefficient

**Figure 3:**
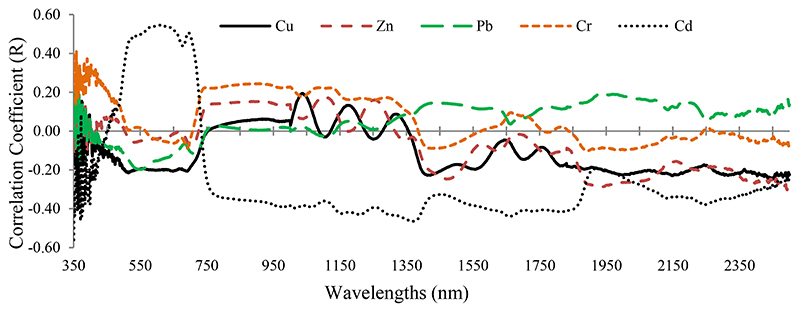
Correlation coefficient between the heavy metal concentration (determined by laboratory analysis) and spectral response of grapevine leaf samples (350 to 2500 nm).

Figure 3 displays the correlation coefficient between grapevine leaves’ reflectance (350–2500 nm) and their heavy metal concentrations (Cu, Zn, Pb, Cr, and Cd). The correlation coefficients were noisiest in the range from 350 to 400 nm due to atmospheric effects. Of particular interest is that the highest absolute correlation coefficient took place in the range of 350 to 400 nm in relation to Cr, Pb, and Zn. Cd showed the best correlation with the wavelengths in the VIS region (400–680 nm), while it dropped sharply in the RDE region (680–750 nm) (Figure 3). This suggests that the RDE region is one of the best options for introducing optimal wavelengths to estimate Cd concentrations in grapevine leaves. Also, the other heavy metals caused subtle fluctuations in the RDE region, and their correlation coefficients tended to be positive. This finding indicates the potential of this region to forecast metal contents in the grapevine leaves. Similar correlation coefficients were observed for Cu, Zn, Cr, and Pb in the NIR spectrum region (750–1300 nm), but Cd had a varied correlation curve in this range. In the MIR region (2500–1300 nm), the heavy metal correlation coefficients were closer together (Figure 3). With the exception of Pb, the remaining metals were negatively correlated with most wavelengths of this region.

In comparison to a related study by Zhuang [41], a similar correlation graph between spectral response (400–2500 nm) and heavy metal contents (Cu, Zn, Pb, Cd, As, and Fe) was obtained. A comparison of Figure 3 with the study results of Zhuang [41] shows that the correlation pattern between the heavy metal contents and the spectral response is not alike. Therefore, the structural and biochemical differences between the studied species (grapevine and rice) and the level of spectroscopy (leaf or canopy level) can be considered as the most important drivers justifying these differences.

### 3.3 Optimal Feature Selection

**Figure 4:**
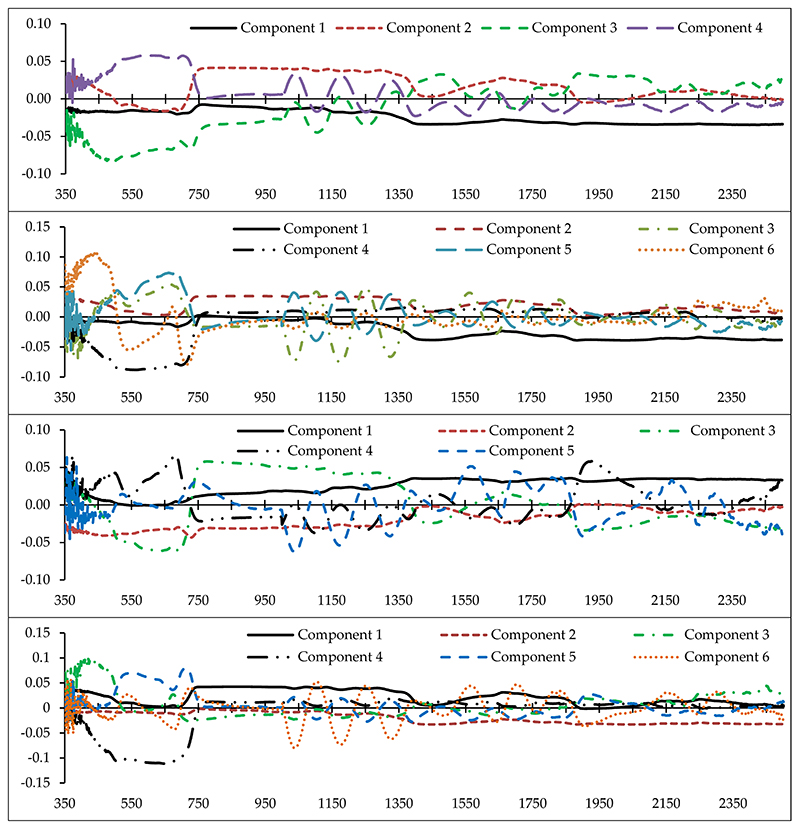
The factor load of wavelengths (350–2500 nm) in the optimal components extracted by the the partial least squares (PLS) method for estimating heavy metal concentrations (from top to bottom) in the grapevine leaves (vertical axis is the factor load).

Determining the optimal wavelengths to monitor the desired plant parameters within the vast hyperspectral bands is one of the most critical operations in spectroscopy [43,46,56]. Commonly, a small number of wavelengths/spectral indices are selected with maximum performance for the study purpose, while missing data should be minimal [46,68]. Thus, we chose the PLS method because of its high adaptability with hyperspectral data to recognize optimal predictive variables (wavelengths and spectral indices) for estimating heavy metals in grapevine leaves [42]. Identifying the fit number of components is one of the most imperative factors in applying the PLS results because the number of components can directly determine the number of model input variables. Accordingly, the cross-validation algorithm was applied to optimize the number of PLS components [43], and then the optimum variable for each of the components was identified. Figure 4 shows the number of optimal components and the wavelength factor loads of the metals studied. This figure shows that the numbers of developed fit components were 4, 6, 5, 6, and 4, for Cu, Zn, Pb, Cr, and Cd, respectively. Therefore, based on the introduced components, the wavelengths and spectral indices which had the highest correlation with the components were identified. They can be subsequently used as optimal spectral wavelengths and indices in the relevant modelling process, especially for estimating metal concentrations in the grape leaves [43].

As shown in Figure 4, the wavelengths in the vicinity of 2431, 809, 489, and 616 nm can be recognized as an optimal rate for estimating Cu content in grapevine leaves. In the same method, wavelengths in the vicinity of 2032, 883, 665, 564, 688, and 437 nm; 1865, 728, 692, 683, and 356 nm; 863, 2044, 415, 652, 713, and 1036 nm; and 1373, 631, 744, and 438 nm were the optimal wavelengths for estimating Zn, Pb, Cr, and Cd, respectively. Based on these results, the VIS, RDE, NIR, and MIR regions introduced eight, eight, three, and five wavelengths for estimating the studied heavy metals, respectively. The most delicate regions to estimate the studied heavy metals in the grapevine leaves were RDE and VIS (particularly the blue region). Consistent with this finding, Liu et al. [38] and Zhuang [41] also reported that VIS and RDE delivered the most optimal wavelengths for estimating heavy metal contents. Moreover, according to the results, the RDE was one of the most influential regions in introducing optimal wavelengths for estimating the contents of Zn, Pb, Cr, and Cd. In confirmation with this finding, Gu et al. [36] noted the RDE region as being sensitive to estimate the variances of metal contents (especially Cd). They suggested the wavelength of 782 nm as an optimal wavelength for estimating Cd concentration in *Brassica rapa* leaves.

In the same way, the optimal spectral indices for estimating contents of Cu, Zn, Pb, Cr, and Cd were also determined based on the interpretation of the PLS results. In Table 2, a summary of the PLS results is presented, which is used to determine the optimal indices to estimate the heavy metal concentrations. As an optimal index for the estimation of Pb, Cr, and Cd concentrations, the Structure Intensive Pigment Index (SIPI)( (proposed by Penuelas et al. [69]), which represents the ratio of carotenoids to chlorophyll, was the most frequent index among the studied indices. Furthermore, the Disease Water Stress Index (DWSI) and Moisture Stress Index (MSI) indices, which are sensitive to water levels in vegetation (water stress), were identified as optimal indices for estimating Zn–Pb and Cu–Cd, respectively. It is worth remarking that the Normalized Difference Vegetation Index (NDVI) was not chosen as the optimal index to predict the studied metal contents. On the other hand, according to Zhuang [41], the NDVI band ratios were extremely useful in monitoring the contents of metals in the paddy canopy. Therefore, it can be argued that, in addition to the structural and biochemical differences between grapevine and paddy species, the differences in studied spectral indices are another reason for differences in the optimal spectral indices.

### 3.4 Modelling and Accuracy Assessment

After determining the optimal spectral wavelengths and indices, two regression approaches— i.e., MLR and SVM—were applied to model the relationships between spectral data and heavy metal concentrations. Table 3 illustrates the best-developed models and validation results using the SVM algorithm. Based on this table, the RBF function was selected as the optimal central function in 60% of the developed models, followed by the linear function (30%). These two functions were therefore considered as the optimal functions for relevant modelling in the studied grapevine leaves.

Table 4 shows the modelling results using the MLR method. In cases where the Durbin–Watson coefficient ranged from 1.5 to 2.5, there was a lack of self-correlation between error terms in the regression model that included 60% of the presented models [40]. However, in relation to the presented models for Pb–Cd (based on wavelengths) and Cr–Cd (based on spectral indices), the Durbin–Watson coefficient was less than 1.5 and lacked one of the most important conditions for using regression modelling. VIF was also considered for the multicollinearity checking between the predictor variables in the regression models. According to Table 4, there was serious multicollinearity (some predictor VIFs exceeded the critical threshold of 10) in the Pb-based-wavelength and Zn-based-spectral index models. Therefore, these models violate the key assumption of multiple linear regression, making these models invalid.

#### 3.4.1 Modelling of Cu Concentration

**Figure 5:**
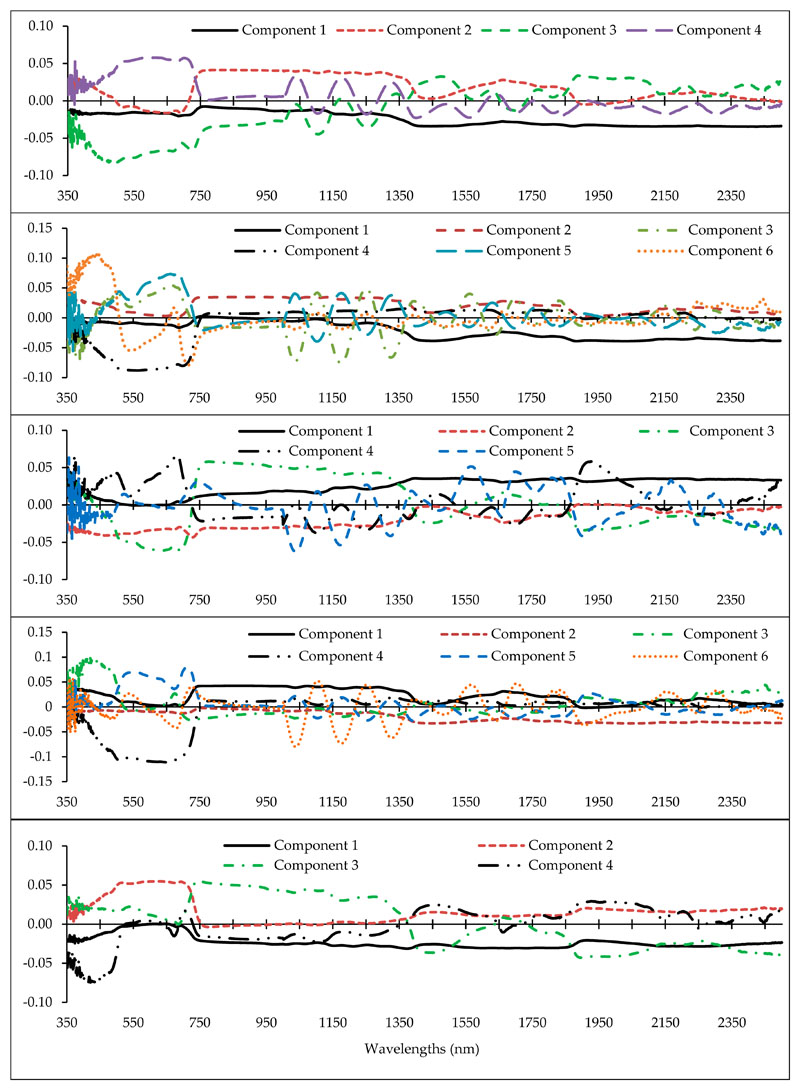
Standardized values (between 0 and 1) of the observed (horizontal axis) and the predicted (vertical axis) concentration of Cu based on wavelengths (top) and spectral indices (bottom) in the testing sets of the SVM and MLR methods.

Figure 5 illustrates the distribution of the observed vs. predicted concentration of Cu in the test set. In some cases, the predicted values were significantly lower than the observed values, which led to a sharp decrease in their accuracy. The optimal wavelengths in the SVM and MLR approaches can predict test samples with 54 and 56% accuracy, respectively. Hence, as a general finding, using wavelengths has a more acceptable performance as opposed to using spectral indices for estimating Cu concentration in the grapevine leaves. In relation to the modelling approaches, it should be noted that, although MLR yielded a slightly superior R^2^ than SVM (at the test set), the SVM–RMSE (25.06) was lower than the MLR–RSME (25.65 mg/kg); therefore, the SVM’s performance seems more acceptable (see also Tables 3 and 4).

#### 3.4.2 Modelling of Zn Concentration

**Figure 6:**
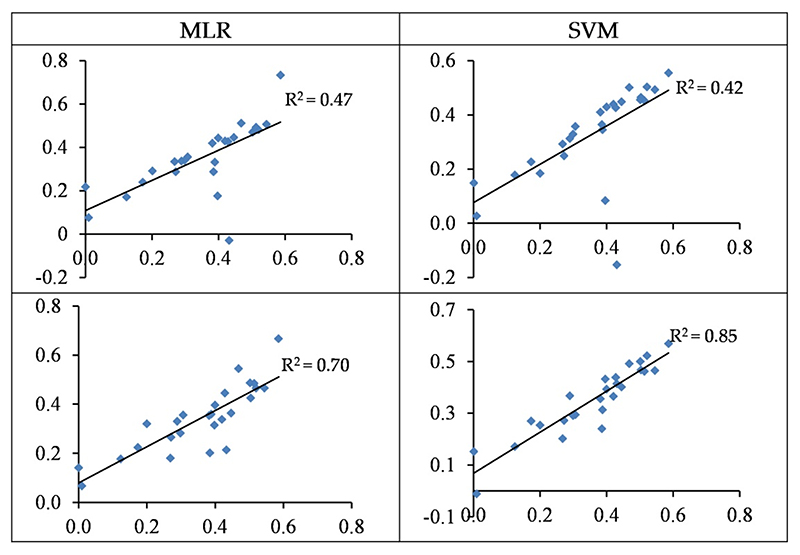
Standardized values (between 0 and 1) of the observed (horizontal axis) and the predicted (vertical axis) concentration of Zn based on wavelengths (top) and spectral indices (bottom) in the testing sets of the SVM and MLR methods.

The SVM and MLR approaches based on wavelengths were able to predict the Zn contents with accuracies of 42%–47% and based on spectral indices with accuracies of 70%–85% in the testing set, respectively (Tables 3 and 4). As shown in Figure 6, the predicted values overestimated the observed values in most cases of wavelength-based models. However, a more uniform distribution was found between the observed and predicted values in spectral indices-based models. Therefore, spectral indices-based models tend to be preferred for predicting Zn contents in the grapevine leaves.

#### 3.4.3 Modelling of Pb Concentration

**Figure 7:**
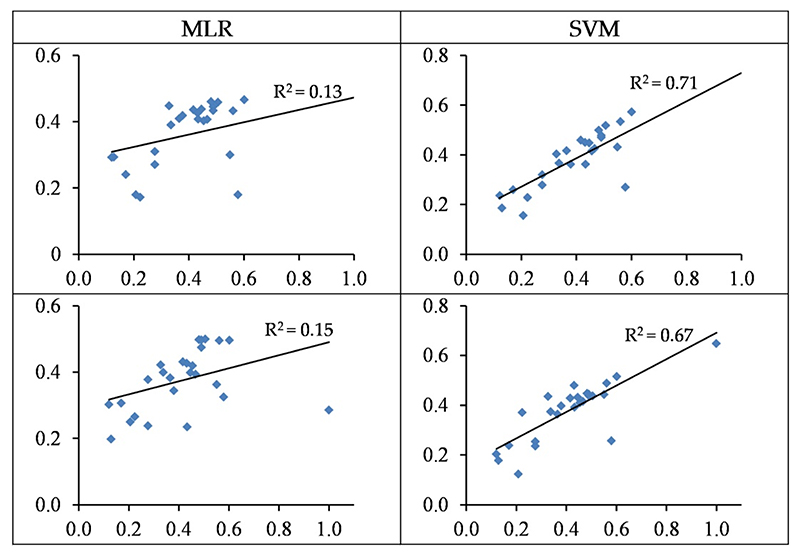
The standardized values (between 0–1) of the observed (horizontal axis) and the predicted (vertical axis) concentration of Pb based on wavelengths (top) and spectral indices (bottom) in the testing sets of the SVM and MLR methods.

The MLR models based on wavelengths and spectral indices yielded a low performance in testing sets with accuracies of 13% and 15%, respectively (Table 4 and Figure 7). Conversely, the SVM model performed more reasonably and predicted Pb contents based on wavelengths and spectral indices in the testing set with accuracies of 71% and 67% (RMSE: 22.49 and 24.51 mg/kg), respectively (Table 3 and Figure 7). It can thus be deduced that SVM is better at estimating Pb contents in the grapevine leaves as opposed to MLR. It should also be noted that the wavelength–SVM model had a more acceptable performance as compared to spectral indices. The obtained results therefore suggest that the wavelength–SVM model is an optimal scenario for estimating Pb contents in grapevine leaves.

#### 3.4.4 Modelling of Cr Concentrations

**Figure 8:**
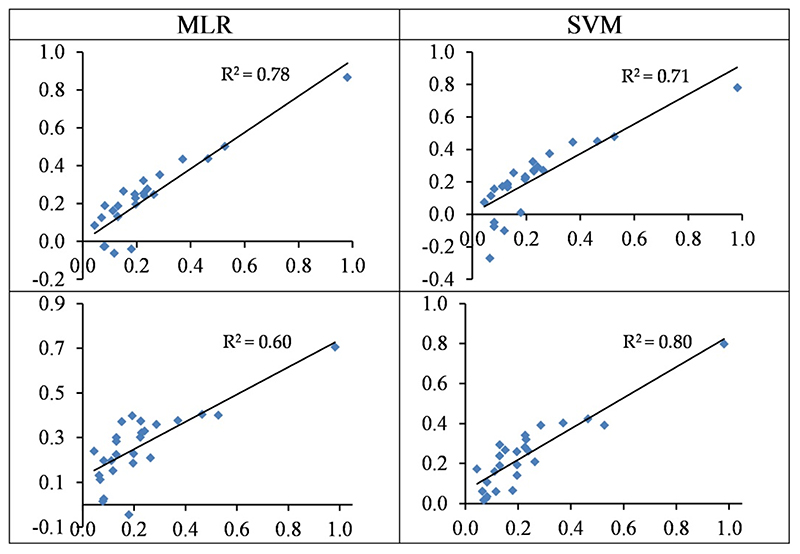
The standardized values (between 0–1) of the observed (horizontal axis) and the predicted (vertical axis) concentration of Cr based on wavelengths (top) and spectral indices (bottom) in the testing sets of the SVM and MLR methods.

Figure 8 shows the distribution pattern of the observed vs. predicted values of Cr in the test set. As shown, the predicted values were overestimated in most cases of MLR, but the predictions of SVM were closer to the observed contents. Overall, the usage of the spectral indices–SVM model was an optimal scenario for estimating Cr contents in the studied grapevine leaves (see also Tables 3 and 4).

#### 3.4.5 Modelling of Cd Concentrations

**Figure 9:**
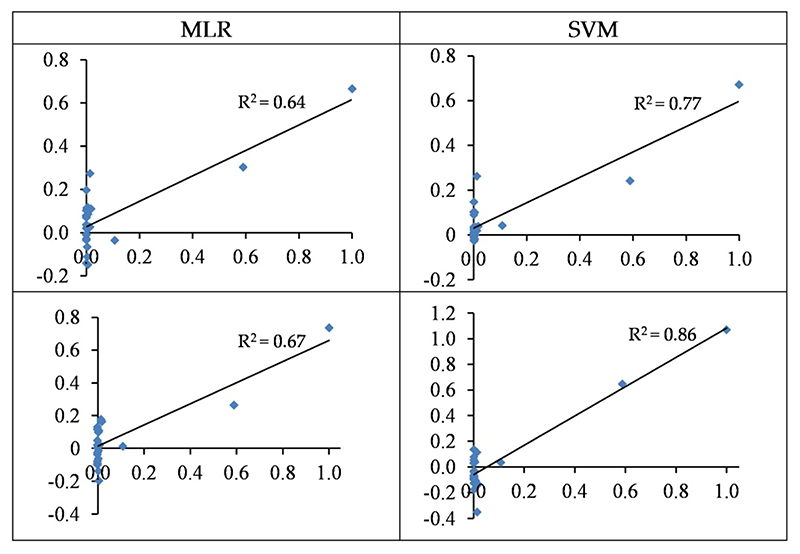
The standardized values (between 0–1) of the observed (horizontal axis) and the predicted (vertical axis) concentration of Cd based on wavelengths (top) and spectral indices (bottom) in the testing sets of the SVM and MLR methods.

The wavelengths-based and spectral indices-based MLR models can estimate Cd contents with accuracies of 64% and 67% in the testing set, respectively (Table 4 and Figure 9). On the other hand, the accuracies of SVM were 77% and 86% in the testing set, respectively. Thus, the SVM outperformed the MLR method at estimating Cd concentrations in the grapevine leaves. It must be admitted, however, that the majority of observed values were around zero, leading to biased estimations. This is also reflected in the RMSE values, which were higher than the other studied metals. Overall, the best model presented by the SVM approach (based on spectral indices) had an RMSE value of 102.85 mg/kg dry weight in the testing set (see also Table 3).

#### 3.5 Summarizing Heavy Metal Modelling

Grapevine leaves are a suitable option for the study of the absorption and accumulation of heavy metals [21]. Therefore, the monitoring of heavy metal concentration can ensure food security as well as the reduction of health and ecological risks [16]. In this study, a stress–stroke method was employed to ensure the appearance of heavy metals in grapevine foliage. This method was also used in similar studies [34,38]. It is important to note that expanding heavy metal masses in plant foliage leads to an increase in the number of reactive oxygen species [70]. Reactive oxygen species are produced in the course of electron transfer activities—mainly in chloroplasts and mitochondria. They also have an important role in consequences such as plant growth retardation, chlorophyll content reduction, inhibition of enzymatic activity, damage to biological molecules (such as lipids, proteins, and nucleic acids, especially DNA), cell membrane peroxidation, and damage to important cellular organelles such as chloroplasts and mitochondria [71,72]. Heavy metal stress, like other non-biotic stresses, leads to changes in the pathways of synthesis of secondary plant metabolites and increases or decreases these compounds [73,74]. It was also observed that heavy metal stress leads to changes in the cuticle position of the leaves and the openings of leaves’ stomas [73]. Considering the effect of heavy metals on the physico-chemical changes in the plant, the spectral pattern of the plant can change, which leads to the spectral pattern differentiation of stressed leaves from healthy leaves. These differentiations can be determined by field-based spectrometry.

According to our results, SVM and MLR prediction methods performed similarly in estimating Cu contents, but in relation to Zn, Pb, Cr, and Cd, the SVM models outperformed the MLR models (Tables 3 and 4). Therefore, the SVM regression method tends to be preferred. Although, in related studies, MLR was the most-used model due to its clarity and structure simplicity [30,41,45], the results of this study recommend SVM for future investigations. The most important reason for the superiority of SVM as opposed to MLR can be attributed to the nature of the relationships between independent and dependent variables. SVM regression was able to perform more accurately in estimating heavy metals due to its high flexibility in training by using both linear and nonlinear functions in the kernel equation [75]. Similarly, a comparison between MLR and artificial neural network (ANN) methods was performed to estimate heavy metals in rice leaves [38]; the results also showed the superior performance of ANN as opposed to MLR.

A comparison between the results obtained for the testing set and the optimal spectral indices and wavelengths in estimating heavy metal contents in various studies was conducted and is shown in Table 5. Based on the R^2^ rate of the test set, the performance order of the presented models was Cd > Zn > Cr > Pb > Cu (Table 5). Therefore, the predictive accuracies for Cd, Zn, and Cu were 86, 85, and 56%, respectively. Li (2011) listed a prediction order accuracy of heavy metals in vegetation as Cr > Pb > Cu > Zn. Furthermore, Zhuang [41] ranked the prediction accuracy of heavy metals in rice as Cu > Pb > Zn, which is different from the findings of the present study (Table 5). The rate of prediction accuracy of Pb in this study is close to the findings of Li [44] and Zhuang [41]. The accuracy of Cr prediction content is also comparable to the results of Li et al. [39]. According to Li [44], Zhuang [41] and Ping et al. [30], Cu predictions were, respectively, 60, 76, and 69%, higher than the present study’s result (56%) (Table 5). However, the present study was able to estimate Zn contents with a higher accuracy compared with the results of Li [44], Zhuang [41], and Kooistra et al. [45], as well as Cd contents as compared to the findings of Ping et al. [30] and Liu et al. [37].

As a final remark, in many studies, RDE and VIS regions were reported to be sensitive to the stress caused by heavy metals [36,38,41]. The comparison of the optimal spectral indices and wavelengths selected for the heavy metal rate predicted in the present study and other related studies show discrepancies (Table 5). The number of spectral samples, spectroscopy acquisition level, spectral range, calculated spectral indices, as well as statistical analyses for data reduction and relationship modelling can all play a role in explaining these differences. Finally, it should also be pointed out that each heavy metal has a special effect, leading to distinct responses depending on the plant species (including leaf colour changes, chlorosis, necrosis, dwarfism, giant, leaf and root spreading, etc.), which can justify this finding [76].

## 4 Conclusion

In this study, we examined the suitability of in-field hyperspectral data (wavelengths from 350 to 2500 nm and 32 spectral indices) in the estimation of heavy metal contents (Cu, Zn, Pb, Cr, and Cd) in vine leaves. Our most important findings are listed as follows:

i) The grapevine’s foliar spectral signatures (reflectance characteristics) altered when applying heavy metal stress due to their effects on the biochemical components and the leaves’ structure. Considerable changes are observed in the VIS, RDE, NIR, and MIR regions of the electromagnetic spectrum.
ii) Significant correlations are found between the heavy metal contents and the grapevine’s foliar spectral response, especially in VIS and RDE regions.
iii) From the reflectance data, 32 spectral indices were formulated using two or more bands. In PLS analysis, it was found that the Simple Ratio (SR), Cellulose Absorption Index (CAI), RATIO9752, and DWSI; R680, Water Index (WI), Lic1, MSI, and Photochemical Reflectance Index (PRI)2; Vogelman Index (VOG), MSI, SIPI, and R550; mNDVI705, Greenness Index (GI), RATIO975, and SIPI; and SIPI and DWSI are more responsive to heavy metal contents compared with the other indices. They are considered to be optimal indices to estimate Cu, Zn, Pb, Cr, and Cd concentrations, respectively.
iv) Also based on the PLS results, the wavelengths in the vicinity of 2431, 809, 489, and 616 nm; 2032, 883, 665, 564, 688, and 437 nm; 1865, 728, 692, 683, and 356 nm; 863, 2044, 415, 652, 713, and 1036 nm; and 1373, 631, 744, and 438 nm are optimal for estimating Cu, Zn, Pb, Cr, and Cd contents in the grapevine leaves, respectively. Accordingly, VIS and RDE emerged as the most sensitive regions for monitoring heavy metal contents in grapevine leaves.
v) In most cases, the SVM regression models yielded more accurate performances when estimating heavy metal contents as opposed to the MLR models. For the best SVM structures, the concentrations of Cu, Zn, Pb, Cr, and Cd are estimated with R^2^ values of 0.56, 0.85, 0.71, 0.80, and 0.86 in the testing set, respectively.
vi) As a general finding, spectral indices yielded more acceptable performance as opposed to wavelengths in forecasting heavy metal contents in the grapevine leaves.

Altogether, the scenario of joining spectral indices with SVM regression is suggested as the most appropriate method for predicting heavy metal contents in the grapevine leaves. At the same time, this conclusion underpins the usage of in-field spectroscopy data and multivariate statistical analysis for the rapid and eco-friendly monitoring of heavy metals in food-producing ecosystems. This study further revealed that the spectral responses of foliar grapevine and other agriculture/horticulture species to heavy metal stress need to be better understood. Similar studies are required to investigate heavy metal spectral signatures in other plant species. Eventually, the ultimate goal of this research line is to integrate field data with spectral data from overpassing aerial and satellite sensors to up-scale and automate the monitoring strategy to the field scale.

## Figures and Tables

**Table 1:** Characteristics of studied hyperspectral indices [13,46].

Indices	Equation	Indices	Equation
Cellulose Absorption Index, CAI	0.5(R2000 + R2200) - R2100	Gitelson and Merzlyak	GM1 = (R750)/(R550)
Moisture Stress Index, MSI	(R1600)/(R820)	Chlorophyll, GM1 and 2	GM2 = (R750)/(R700)
Normalized Difference Water Index, NDWI	(R860 - R1240)/(R860 + R1240)	Lichtenthaler Indices, Lic1 to 3	Lic1 = (R800 - R680)/(R800 + R680)
Disease Water Stress Index, DWSI	(R802 + R547)/(R1657 + R682)		Lic2 = (R440)/(R690)
Band ratio at 975 nm, RATIO975	2×R960 –990/(R920 – 940 + R1090 - 1110)		Lic3 = (R440)/(R740)
Band ratio at 1200 nm, RATIO975-2	2×R1180 - 1220/(R1090 – 1110 + R1265 – 1285)	Simple Ratio Pigment Index, SRPI	(R430)/(R680)
Leaf Chlorophyll Index, LCI	(R850 - R710)/(R850 + R680)	Normalized Phaepophytiniz Index, NPQI	(R415 - R435)/(R415 + R435)
DattA	(R780 - R710)/(R780 – R680)	Normalized Pigment Chlorophyll Ratio Index, NPCI	(R680 - R430)/(R680 + R430)
Modified Red Edge Normalized Difference Vegetation Index, mNDVI705	(R750 +R705)/(R750 + R705 - 2×R445)	Greenness Index, GI	(R554)/(R677)
Chlorophyll Index, SGB	(R750 - R445)/(R705 - R445)	Water Index at 1180nm, WI1180	(R900)/(R1180)
Structure Intensive Pigment Index, SIPI	(R445 - R800)/(R680 - R800)	Normalized Difference Vegetation Index, NDVI	(R831 - R667)/(R831 + R667)
Simple Ratio, SR	(R774)/(R677)	Carter Index, CI	(R760/R695)
Reflectance at 550 nm, R550	(R550)	Vogelman Index, VOG	(R740/R720)
Reflectance at 680 nm, R680	(R680)	Carotenoid Reflectance Index, CRI	R800(1/R520 - 1/R550)
Water Index, WI	(R900)/(R970)	Photochemical Reflectance Index, PRI	PRI1 = (R531 - R570)/(R531 + R570)
			PRI2 = 1.5(R830 - R660)/(R830 - R660 + 0.5)
			PRI3 = (R539 - R570)/(R539 + R570)

R: Reflectance.

**Table 2:** Summary of the PLS results on the number of components and optimal indices for estimating heavy metal contents in grapevine leaves.

Heavy Metal	No. of Optimal Components	Cumulative Variance (%)	Optimal Indices in Components
**Cu**	4	82	SR, CAI, RATIO9752, and DWSI
**Zn**	5	84	R680, WI, Lic1, MSI, and PRI2
**Pb**	4	88	VOG, MSI, SIPI, and R550
**Cr**	4	92	mNDVI705, GI, RATIO975, and SIPI
**Cd**	2	81	SIPI and DWSI

**Table 3:** Modelling and validation results of the best support vector machine (SVM) models based on optimal wavelengths and spectral indices for estimating heavy metal concentrations in grapevine leaves in training and testing sets. RBF: radial basis function.

Hyperspectral Data Type	Heavy Metal	Model Structure					Train		Test	
		Kernel Function	No. of Vectors	Coefficient	Degree	Gamma	R^2^	RMSE*	R^2^	RMSE*
WaveLengths	Cu	RBF	13	-	-	0.25	0.97	7.46	0.54	25.06
	Zn	Linear	25	-	-	-	0.67	22.50	0.42	29.65
	Pb	RBF	21	-	-	0.20	0.89	22.28	0.71	24.09
	Cr	Linear	30	-	-	-	0.84	5.61	0.71	7.82
	Cd	RBF	34	-	-	0.25	0.78	98.16	0.77	103.09
Spectral Indices	Cu	Linear	32	-	-	-	0.88	13.01	0.50	25.46
	Zn	RBF	23	-	-	0.8	0.92	13.42	0.85	15.94
	Pb	RBF	24	-	-	0.4	0.85	22.49	0.67	24.51
	Cr	RBF	43	-	-	0.32	0.80	7.27	0.79	6.11
	Cd	Polynomial	19	1	11	0.7	0.88	91.94	0.86	102.85

* mg/kg: dry weight.

**Table 4:** The results of modelling and validation of the best multiple linear regression (MLR) models based on optimal wavelengths and spectral indices for estimating heavy metals concentrations in grapevine leaves in training and testing sets.

Hyperspectral	Heavy Metal	Predictor Variable VIF	Sig. of Regression	Durbin–	Model Structure	Train		Test	
						R^2^	RMSE*	R^2^	RMSE*
Wavelengths	Cu	All <10	<0.05	2.17	C_Cu_ = –1.27 – (0.28×R2431) + (4.08×R809) – (5.32×R489) – (8.73×R616)	0.94	9.35	0.56	25.60
	Zn	All <10	<0.05	2.18	C_Zn_ = –1.11 – (5.77×R2032) – (1.83×R665) + (2.38×R564) + (13.85×R688) – (7.7×R437)	0.73	20.46	0.47	399.13
	Pb	Some cases >10	>0.05	1.39	C_Pb_ = 0.46 – (5.1×R692) + (6.24×R683)	0.32	25.29	0.13	27.28
	Cr	All <10	<0.05	1.59	C_Cr_ = 0.61 + (18.08×R415) – (1.41×R2044) – (4.01×R652) – (1.99×R1036) + (1.11×R713)	0.84	5.58	0.78	6.79
	Cd	All <10	<0.05	1.38	C_Cd_ = 0.98 + (2.76×R1373) + (3.15×R631) + (1.04×R744) – (5.09×R438)	0.63	132.79	0.64	117.26
Spectral Indices	Cu	All<10	<0.05	1.74	C_Cu_ = –2.95 + (3.38×SR) – (0.01×CAI) + (6.76×RATIO9752) – (0.77×DWSI)	0.89	12.63	0.52	25.33
	Zn	Some cases >10	<0.05	1.81	C_Zn_ = –2.26 – (11.34×R680) + (41.89×WI) + (20.68×Lic1) – (3.63×MSI) – (4.14×PRI2)	0.87	15.73	0.70	20.38
	Pb	All<10	<0.05	1.55	C_Pb_ = 2.53 – (1.33×VOG) + (1.93×MSI) + (0.85×SIPI)	0.50	24.45	0.15	27.03
	Cr	All<10	<0.05	1.06	C_Cr_ = –4.97 + (5.23× mNDVI705) + (0.17×GI) – (1.28×RATIO975)	0.59	8.48	0.60	8.78
	Cd	All<10	<0.05	1.27	C_Cd_ =–6.66 + (4.70×SIPI) + (1.13×DWSI)	0.66	121.77	0.67	112.17

* mg/kg: dry weight, Rn: reflections at a certain wavelength, Cn: concentration of a certain heavy metal.

**Table 5:** Comparison results of the best models presented in this study and other similar studies iπ relation to the estimation of heavy metal contents in plant species using field-based spectrometry.

Metal	Reference	Plant/Species	Approach	Optimal Spectral Indices/Wavelengths	R^2^
Cu	Present study	Grape	MLR	R616, R489, R809, R2431	0.56
	Li [44]	Vegetation	MLR		0.60
	Zhuang [41]	Paddy/Rice	MLR		0.76
	Ping et al. [30]	Maize	MLR	NI15, NI11	0.69
Zn	Present study	Grape	SVM	WI, Lic1, MSI, PRI2 R680	0.85
	Li [44]	Vegetation	MLR		0.48
	Zhuang [41]	Paddy/Rice	MLR	661.96×R2210-136.26	0.34
	Kooistra et al. [45]	Grass	MLR	MSAVI2	0.64
	Liu et al. [37]	Rice	ANN		0.95
Pb	Present study	Grape	SVM	R683, R356 R692, R1865, R728	0.71
	Li [44]	Vegetation	MLR		0.77
	Zhuang [41]	Paddy/Rice	MLR		0.70
	Ping et al. [30]	Maize	MLR	NI15, NI17	0.87
Cr	Present study	Grape	SVM	mNDVI705, GI, RATIO975, SIPI	0.80
	Ping et al. [30]	Maize	MLR	NI5, R553	0.49
	Li et al. [39]	Vegetation	MLR	R688, R672, R874, R677, R678, R679, R 680, R566	0.81
Cd	Present study	Grape	SVM	SIPI, DWSI	0.86
	Ping et al. [30]	Maize	MLR	NI11, NI17	0.63
	Liu et al. [37]	Rice	MLR		0.70

NI11: (R700–R690)/(R700+R690), NI15: (R760–850–R350–400)/(R760–850+R350–400), NI17:(R1220– R510)/(R1220+R510), R*n*: Reflections at a certain wavelength.
